# SRRM2 may be a potential biomarker and immunotherapy target for multiple myeloma: a real-world study based on flow cytometry detection

**DOI:** 10.1007/s10238-023-01272-1

**Published:** 2024-01-30

**Authors:** Jinjing Guo, Zhiye Zhang, Huiping Wang, Qian Li, Mengmeng Fan, Wanqiu Zhang, Qianshan Tao, Zhitao Wang, Chun Ling, Hao Xiao, Zhimai Gao, Zhimin Zhai

**Affiliations:** 1 Department of Hematology, Hematological Research Center, The Second Affiliated Hospital of Anhui Medical University, Hefei, Anhui China; 2 Department of Laboratory, Fuyang People’s Hospital, Fuyang, China; 3 Department of Hematology, Fuyang Hospital Affiliated to Anhui Medical University, Fuyang, China; 4 Department of Hematology, Affiliated Chuzhou Hospital of Anhui Medical University, Chuzhou, Anhui China; 5 ZENO Biotechnology (Shenzhen) Co, Shenzhen, Guangzhou, China

**Keywords:** Multiple myeloma, Serine/arginine repetitive matrix 2 (SRRM2), Immunotherapeutic target, Biomarker

## Abstract

Serine/arginine repetitive matrix 2 (SRRM2) has been implicated in tumorigenesis, cancer development, and drug resistance through aberrant splicing; however, its correlation with multiple myeloma (MM) has not been reported. We investigated the potential of SRRM2 as a biomarker and immunotherapeutic target in MM by examining its expression in MM cells using flow cytometry. Our study included 95 patients with plasma cell disease, including 80 MM cases, and we detected SRRM2 expression on plasma cells and normal blood cells to analyze its relationship with clinical profiles. We found widespread positive expression of SRRM2 on plasma cells with little expression on normal blood cells, and its expression on abnormal plasma cells was higher than that on normal plasma cells. Comparative analysis with clinical data suggests that SRRM2 expression on plasma cells correlates with MM treatment response. MM patients with high SRRM2 expression had higher levels of serum β2-mg and LDH, ISS staging, and plasma cell infiltration, as well as high-risk mSMART 3.0 stratification and cytogenetic abnormalities, particularly 1q21 amplification. In patients with previous MM, high SRRM2 expression on plasma cells was associated with higher plasma cell infiltration, high-risk mSMART 3.0 risk stratification, cytogenetic abnormalities, more relapses, and fewer autologous stem cell transplant treatments. In summary, SRRM2 may serve as a novel biomarker and immunotherapeutic target for MM. Its expression level on plasma cells can help in risk stratification of MM and monitoring of treatment response.

**Supplementary Information:**

The online version contains supplementary material available at 10.1007/s10238-023-01272-1.

## Introduction

Multiple myeloma (MM) is a terminally differentiated B-cell tumor and is the second most common hematologic malignancy [1–3]. In recent years, the application of proteasome inhibitors, immunomodulation, and immunotherapy has led to significant advances in the treatment of first-line and relapsed refractory (R/R) MM, greatly improving patient objective response rates (ORR) and durable complete remission rates (CR) [4]. In particular, immunotherapies, including CD38 monoclonal antibody and BCMA Chimeric antigen receptor (CAR) T cell therapy, have shown stunning results in patients with MM [5, 6]. However, MM can still recur incurably. Due to the limited immunotherapy targets available for MM. Furthermore, the loss of immune therapy activity occurs through various mechanisms, such as antigen shedding, interference with soluble ligands, or even gene deletion, resulting in antigen loss. Therefore, expanding immune therapy targeting the repertoire is crucial for salvaging therapy after relapse. At the same time, a multi-targeted combination can provide a broader antigen coverage and thus improve treatment efficacy [7, 8]. Likewise, new drugs and immunotherapy bring new changes to the prognosis of MM and need to be supplemented with more diagnostic, risk stratification, and prognostic markers for clinical application [9].

The serine/arginine repetitive matrix 2 (SRRM2) protein is located in the nucleus of normal human cells and is rich in serine/arginine (RS) repeats. It can be found by cryo-electron microscopy (EM) in spliceosomal structures with subcellular structures localized within Cajal vesicles and nuclear spots (NS), acting mainly in selective splicing of cells and involved in mRNA pre-maturation [10, 11]. Research has shown that SRRM2 can regulate the ATF4-serine metabolic pathway and affect the vitality, proliferation, and differentiation of tumor cells. THP-1 cells with SRRM2 gene knockout exhibited increased sensitivity to camptothecin (CPT) and azacitidine (AZA) while significantly downregulating the expression of MUC1 (mucin-1). MUC1 plays an important role in the development, metastasis, and anti-apoptotic processes of cancer [12]. In addition, there are data sets showing that increased expression of SRRM2 is associated with poor survival in acute myeloid leukemia, renal, and hepatocellular carcinoma (proteinatlas.org, BloodSpot). To date, there are no reports on the correlation between SRRM2 and MM. This study aimed to use flow cytometry to detect the expression of SRRM2 on plasma cells in plasma cell disorders, particularly MM, and to explore whether SRRM2 protein can serve as a biomarker for MM and a potential target for immunotherapy.

## Materials and methods

### Patients and samples

This study was approved by the Medical Research Ethics Committee of the Second Affiliated Hospital of Anhui Medical University. A total of 95 patients with clinically suspected or confirmed plasma cell dyscrasias at the Second Affiliated Hospital of Anhui Medical University from January 2022 to February 2023 were included in the analysis. The enrolled patients included 7 with reactive plasmacytosis, 80 with MM, and 8 with other plasma cell disorders. Other plasma cell disorders included 3 cases of immunoglobulin light chain amyloidosis, 1 case of extramedullary plasmacytoma, 2 cases of monoclonal immunoglobulin disease, and 2 cases of immunoglobulin deposition disease that were not excluded. Of the 80 patients with MM, 35 were newly diagnosed, and 47 had a previous diagnosis of regular admission, including plasma cell leukemia (PCL). The diagnosis of MM was based on the SLiM CRAB criteria in accordance with the International Myeloma Working Group (IMWG) guidelines [13, 14]. Staging was performed according to the Durie–Salmon staging system, the International Staging System, and the Revised International Staging System, based on clinical testing for MM [15–17]. The mSMART 3.0 risk stratification for MM was performed based on cytogenetic FISH analysis [18]. Table S1 summarizes the baseline characteristics of the 95 patients.

**Fig. 1:**
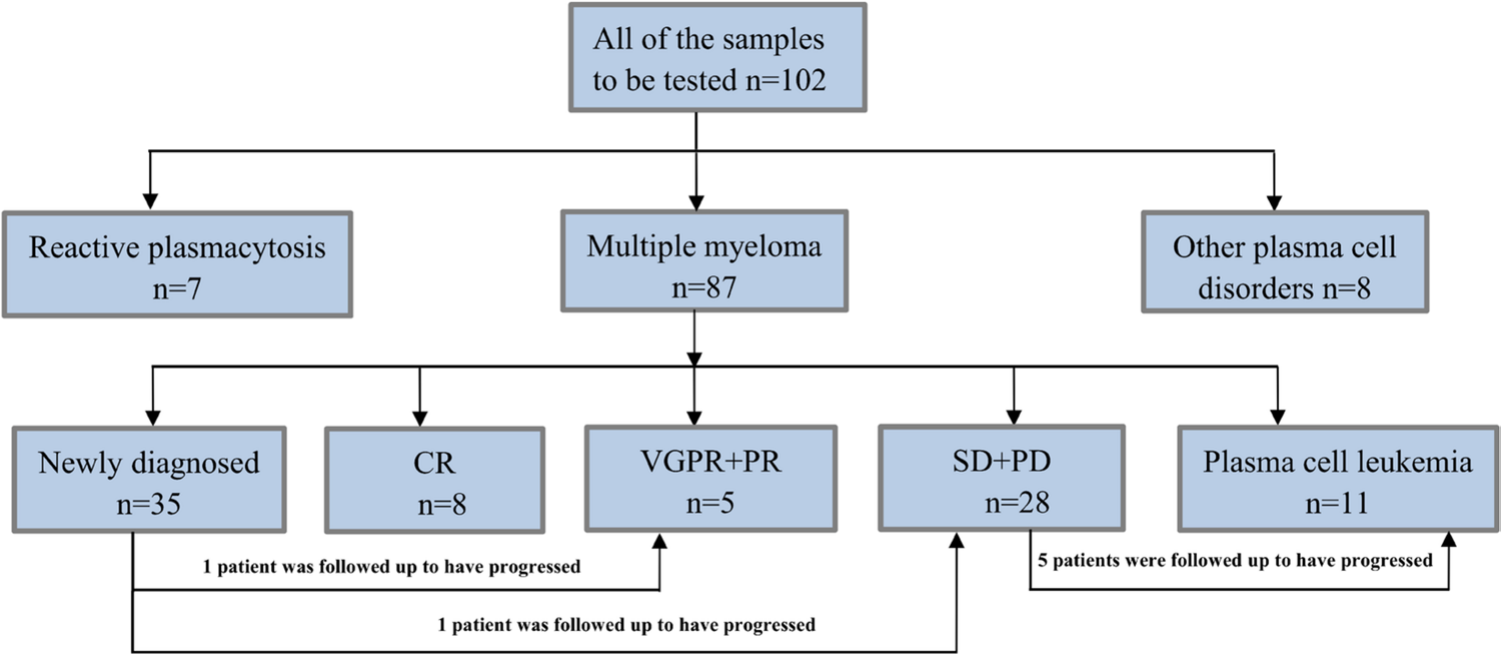
Structured diagram of the patient’s clinical diagnosis and follow-up tests. A total of 102 samples from 95 patients were analyzed, including 7 patients with reactive plasmacytosis, 80 patients with MM, and 8 patients with other plasma cell disorders. The 87 MM samples were divided into five subgroups based on assessment of response to treatment and follow-up testing of MM patients. In the treatment follow-up evaluation, one patient with newly diagnosed MM entered the PR stage and one progressed to the PD stage; five patients with PD stage progressed to PCL

A total of 102 samples were tested in 95 patients, including 87 bone marrow and 15 peripheral blood samples. Figure 1 illustrates the clinical diagnosis and follow-up testing of all patients. A total of 87 samples were tested in the MM group, including 35 samples from patients with newly diagnosed MM (NDMM), 11 samples from PCL, and 41 samples from patients on treatment. Of these, the on-treatment patients were evaluated for complete response (CR), partial response (PR), very good partial response (VGPR), stable disease (SD), and disease progression (PD) according to IMWG guidelines. The plasma cells detected in samples from the CR subgroup of 8 cases of MM were normal phenotype plasma cells. Among all samples, there was one peripheral blood sample in the reactive plasmacytosis group, three peripheral blood samples in the PD subgroup of the MM group, and all PCL subgroup samples were peripheral blood, while all other samples were bone marrow samples.

### MM cell lines

The human MM cell lines RPMI-8226, U226, H929 were cultured in RPMI 1640 medium (Hyclone, Logan, UT, USA) supplemented with 10% heat-inactivated fetal bovine serum (FBS; EVERY GREEN Zhejiang Tianhang Biotechnology Co. Ltd., China) and 1% antibiotics (100 U/mL penicillin G and 100 mg/mL streptomycin). All cells were conserved in our laboratory and cultured in a cell incubator (Thermo Fisher Scientific Inc. USA) containing 5% CO2 at 37 °C. All cell lines were authenticated for immunophenotyping prior to the experiments.

### Immunohistochemical testing

Bone marrow biopsy samples were obtained from MM patients with typical plasma cell infiltration. SRRM2 immunohistochemical staining was performed on the biopsy section, along with isotype control staining and hematoxylin and eosin (HE) staining. The following are the experimental procedures.

Cut the bone marrow tissue wax blocks into 3μm sections and place them on slides, bake at 65°C for 60 min, and then dewax in xylene and hydrate with graded ethanol. Soak the hydrated tissue sections in citrate buffer (pH 6.0) and perform antigen retrieval in a pressure cooker. After cooling the sections to room temperature, block endogenous peroxidase with 3% H2O2, stain with a 1:2000 dilution of a monoclonal antibody against human SRRM2 (rat-derived, same-species specific), and incubate at 37°C for 1 h. After primary antibody incubation, add a peroxidase-labeled goat anti-mouse/rabbit IgG polymer (immunostaining kit; CELNOVTE Henan Celnovte Biotechnology Co., Ltd., China) and incubate at 37°C for 30 min; finally, perform counterstaining with hematoxylin. Typical fields (40 ×) were selected under a microscope and analyzed by two pathologists for confirmation.

### Sample preparation for flow cytometry analysis

Heparin-anticoagulated bone marrow and peripheral blood specimens were collected, and two tubes were labeled in parallel for each specimen. Adjust the number of nucleated cells in each tube to 5–8 × 10^5^. Add mouse anti-human SRRM2-specific recombinant antibody and matching isotype control antibody to two parallel tubes and incubate with all cells in the sample. After incubation at room temperature for 30 min, add 1.5 ml of saline and wash 3 times, centrifuge at 300 g/5 min and resuspend in 200 μL saline. Goat anti-rat IgG polyclonal to FITC fluorescent antibody (Abcam, batch ab150165, Cambridge, UK) and other fluorescent-labeled monoclonal antibodies (CD138-PE, CD38-APC,CD45-PC7, Beckman Coulter, Miami, FL, USA) were added, incubated for 15 min, and then treated with NH4Cl for 10 min for flow cytometry analysis. For the detection of all clinical samples, we uniformly used a flow cytometry protocol with four-color fluorescent labeling (FITC, PE, APC, and PC7) to analyze the expression of SRRM2 in tumor cells and other normal blood cells. Data acquisition and analysis were performed using a flow cytometer (Cytoflex S, Beckman Coulter) and CytExpert software (Beckman Coulter). A minimum of 10^5^ nucleated cells and 500 target cells were obtained for most samples. For samples with fewer target cells, we obtained more cells by centrifugation or extended collection time.

### Statistical analysis

The comparison between two groups was performed using t tests, Welch’s t test, and Wilcoxon rank sum nonparametric test. The overall comparison of multiple groups was performed using one-way ANOVA test, Welch’s one-way ANOVA test, and Kruskal–Wallis test, as well as Dunn’s test. A ROC curve was constructed to evaluate the sensitivity and specificity of SRRM2 in distinguishing between normal and abnormal plasma cells, as well as in the diagnosis of newly diagnosed MM. The differences between SRRM2 expression and clinical characteristics of patients were analyzed using Pearson chi-square test or Fisher’s exact test for categorical variables, and Mann–Whitney U test or Student’s t test for continuous variables. A line graph was used to determine the trend of SRRM2 changes, and a one-way repeated measures ANOVA was employed. The data were processed using SPSS v.24.0 software and R (4.2.1) software, and the ggplot2 package was used for data visualization. Two-sided *p* values ≤ 0.05 indicated statistical significance.

## Results

### Flow cytometry protocols

**Fig. 2:**
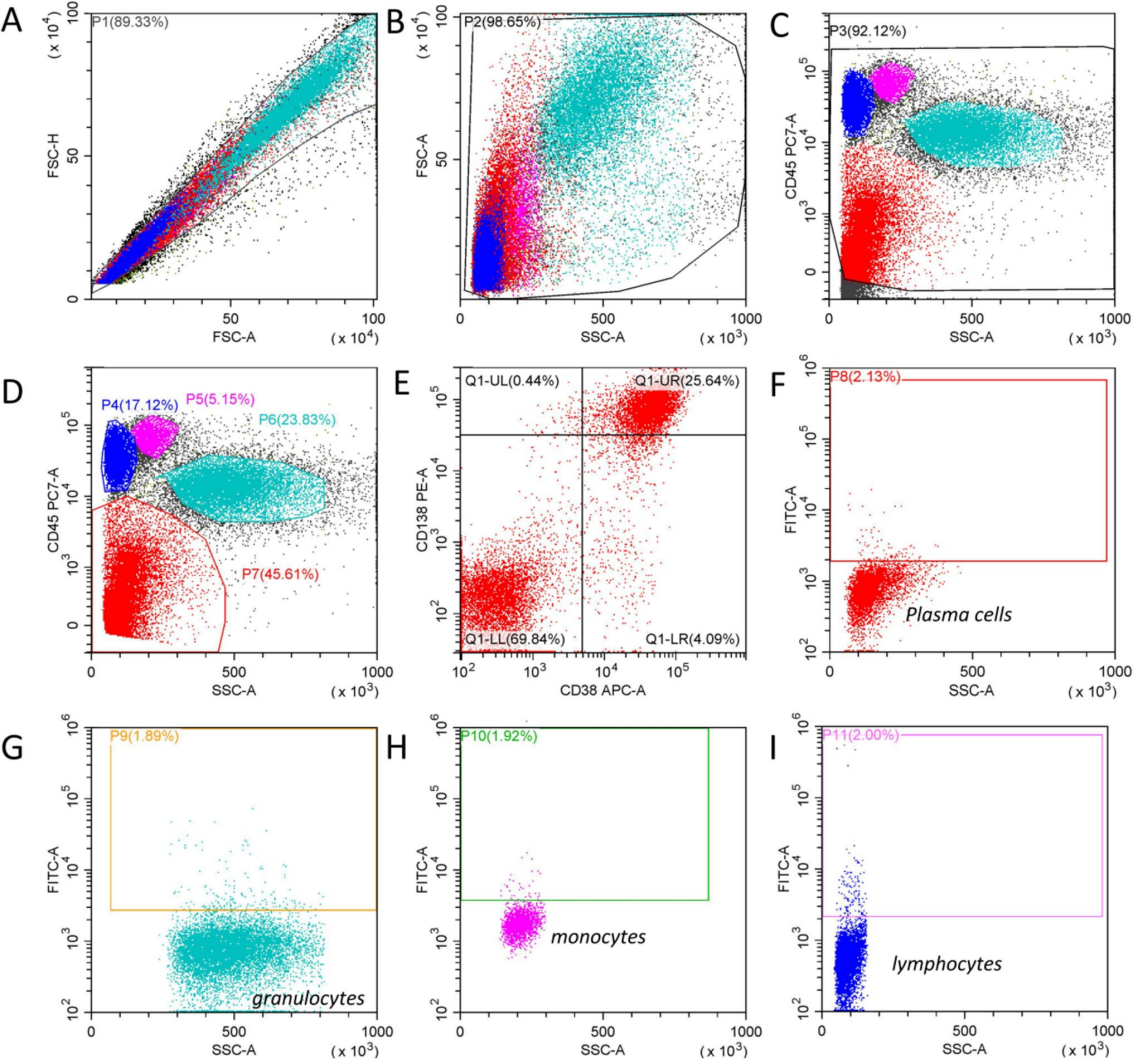
Isotype controls were used to determine the negative cutoff values for each cell population (Tube 1). **A** All nucleated cells were gated P1 after removing adhesion cells and impurities. **B** P1 was plotted on a FSC versus SSC display with cell debris removed. The remaining cells were gated P2. **C** P2 was plotted on a CD45 versus SSC display, further removal of cell debris and impurities. The remaining cells were gated P3. **D** Granulocytes, lymphocytes, and monocytes were gated within the gates of P6, P4, and P5, respectively, and dim expression of the CD45 antigen cells was gated within the gate of P7. **E** P7 was plotted on a CD38/CD138 display. The plasma cells (Q1-UR) were then identified by positive expression of CD38 and CD138. **F** Q1-UR were plotted on a ISO-FITC/SSC display to determine the negative boundary for plasma cells and nonspecifically positive isotype Control cells were gated P8. **G, H, I** P6, P5, and P4 were plotted on a ISO-FITC/SSC display, respectively, to determine the negative boundaries for granulocytes, lymphocytes, and monocytes

**Fig. 3:**
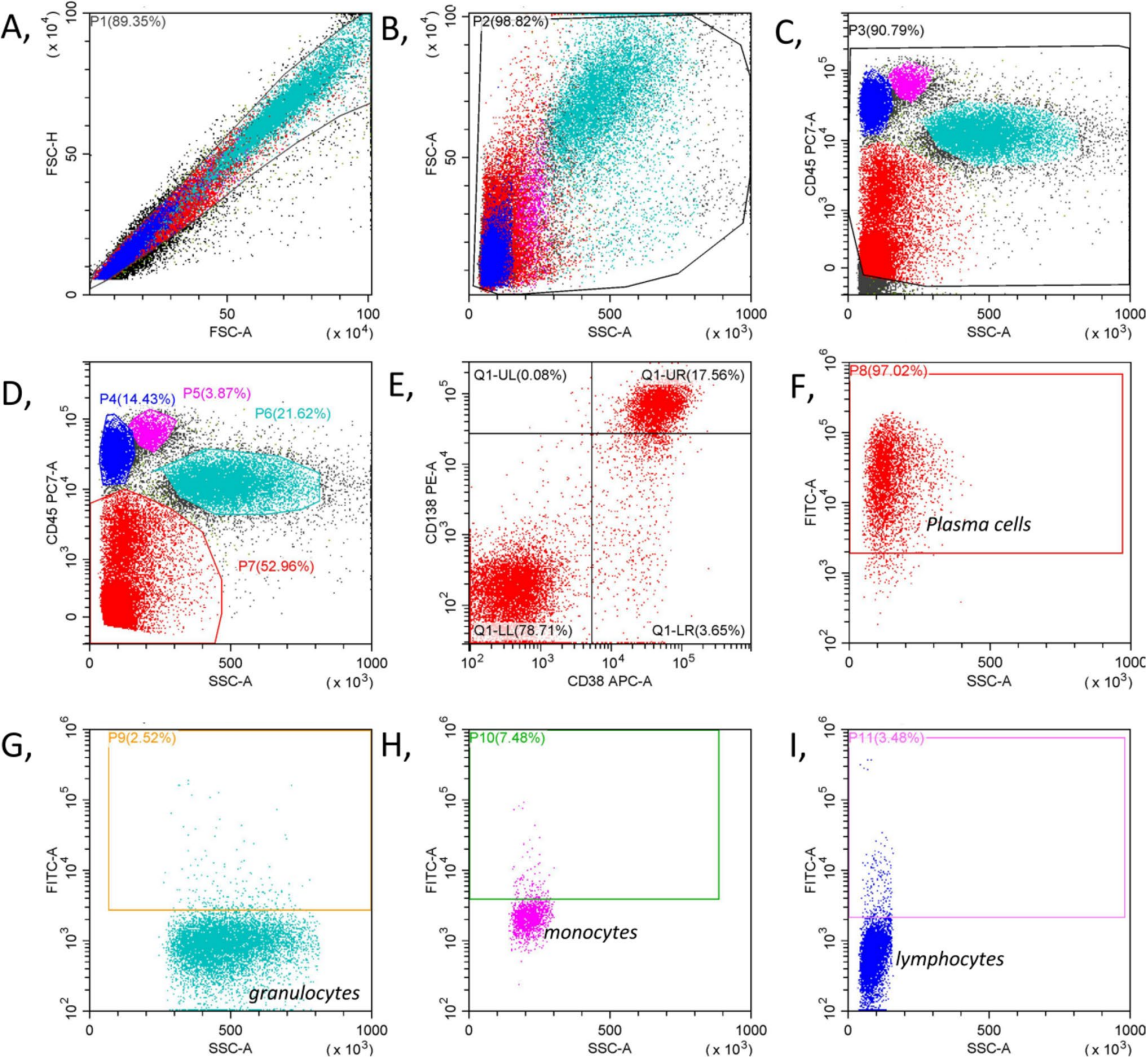
SRRM2 antibody staining tubes were used to determine the percentage of positivity for each cell population using ISO as a baseline (Tube 2). **A,** All nucleated cells were gated P1 after removing adhesion cells and impurities. **B,** P1 was plotted on an FSC versus SSC display with cell debris removed. The remaining cells were gated P2. **C,** P2 was plotted on a CD45 versus SSC display, further removal of cell debris and impurities. The remaining cells were gated P3. **D,** Granulocytes, lymphocytes, and monocytes were gated within the gates of P6, P4, and P5, respectively, and dim expression of the CD45 antigen cells was gated within the gate of P7. **E,** P7 was plotted on a CD38/CD138 display. The plasma cells (Q1-UR) were then identified by positive expression of CD38 and CD138. **F,** Q1-UR were plotted on a SRRM2-FITC/SSC display. The negative cutoff value of tube 1 (ISO) was used as baseline to determine the expression of SRRM2 on plasma cells, and positive cells were gated P8. **G,** **H,** **I,** P6, P5, and P4 were plotted on a SRRM2-FITC/SSC display, respectively, to determine the percentage of cells positive for SRRM2 expression according to the negative cutoff set by tube 1 (ISO)

**Fig. 4:**
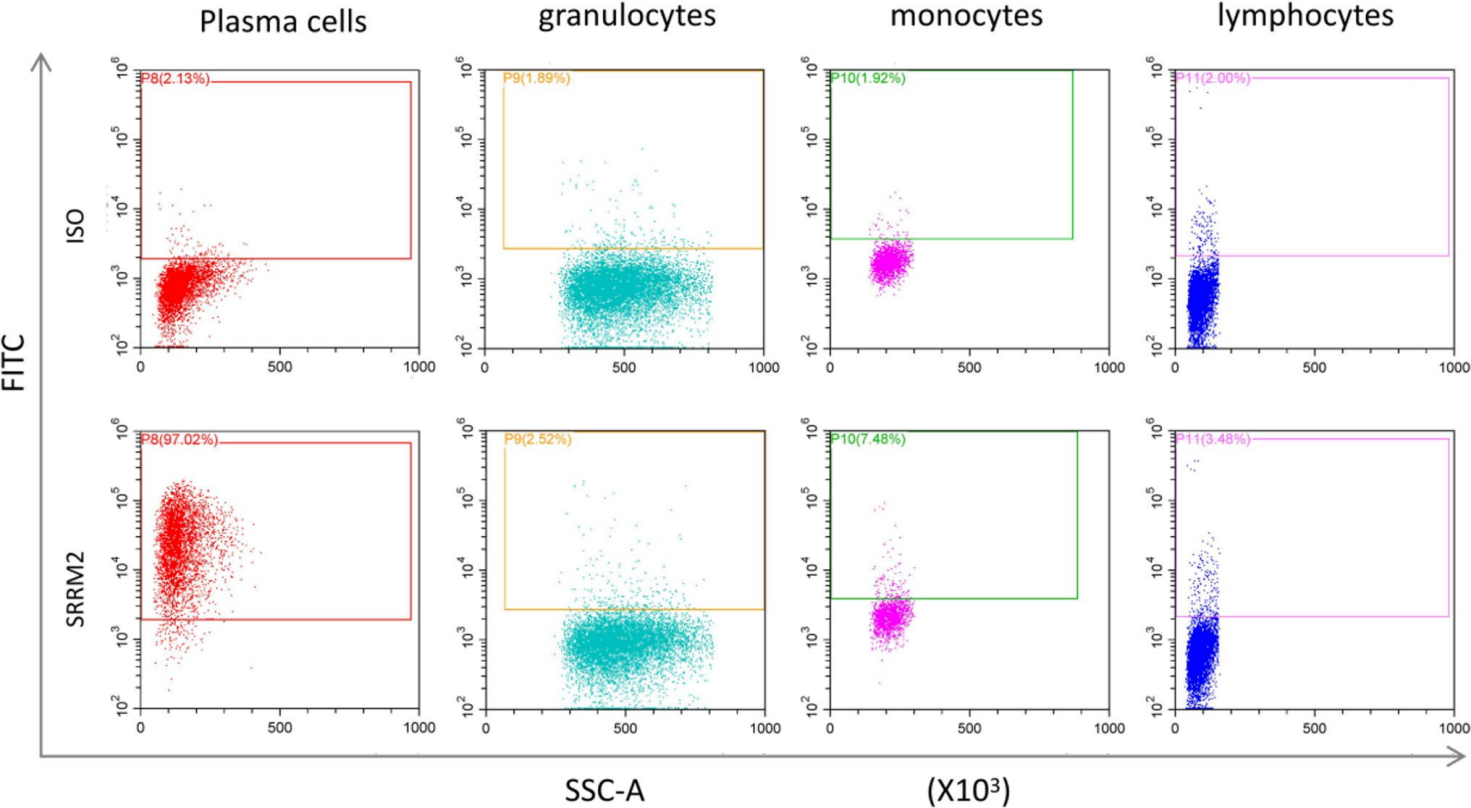
Compare and analyze the percentage of positive expression of various cell populations in the test tube using the homotypic control as the negative threshold Isotype control (ISO) expression at approximately 2% in plasma cells, granulocytes, monocytes, and lymphocytes in test tube 1 served as the baseline for assessing SRRM2 expression in the same cells in test tube 2. The percentage of SRRM2 expression in test tube 2 was determined with reference to the negative threshold established by ISO in test tube 1 for each cell type

To optimize the use of fluorescent antibodies as much as possible, a uniform four-color flow cytometry protocol was designed, and plasma cell populations were identified and gated by CD45-PC7, CD138-PE, and CD38-APC. Granulocyte, monocyte, and lymphocyte populations were localized and gated by CD45-PC7 and SSC. The percentage and mean fluorescence intensity of SRRM2-FITC expression on plasma cells and normal blood cells, respectively, were analyzed. In two parallel tubes for each test sample, the expression of approximately 2% FITC for each cell population in the isotype control tube was used to define the negative cutoff value for this cell population (Fig. 2). The percentage of SRRM2 expression in each cell population was calculated by comparing each cell population in the experimental tube to the negative cutoff value set by the isotype control tube (Figs. 3, 4). To minimize the effect of non-specific reactions, we defined a positive result when ≥ 30% of cells in any cell population in the experimental tube expressed SRRM2.

### SRRM2 is positively expressed on multiple myeloma plasma cells

**Fig. 5:**
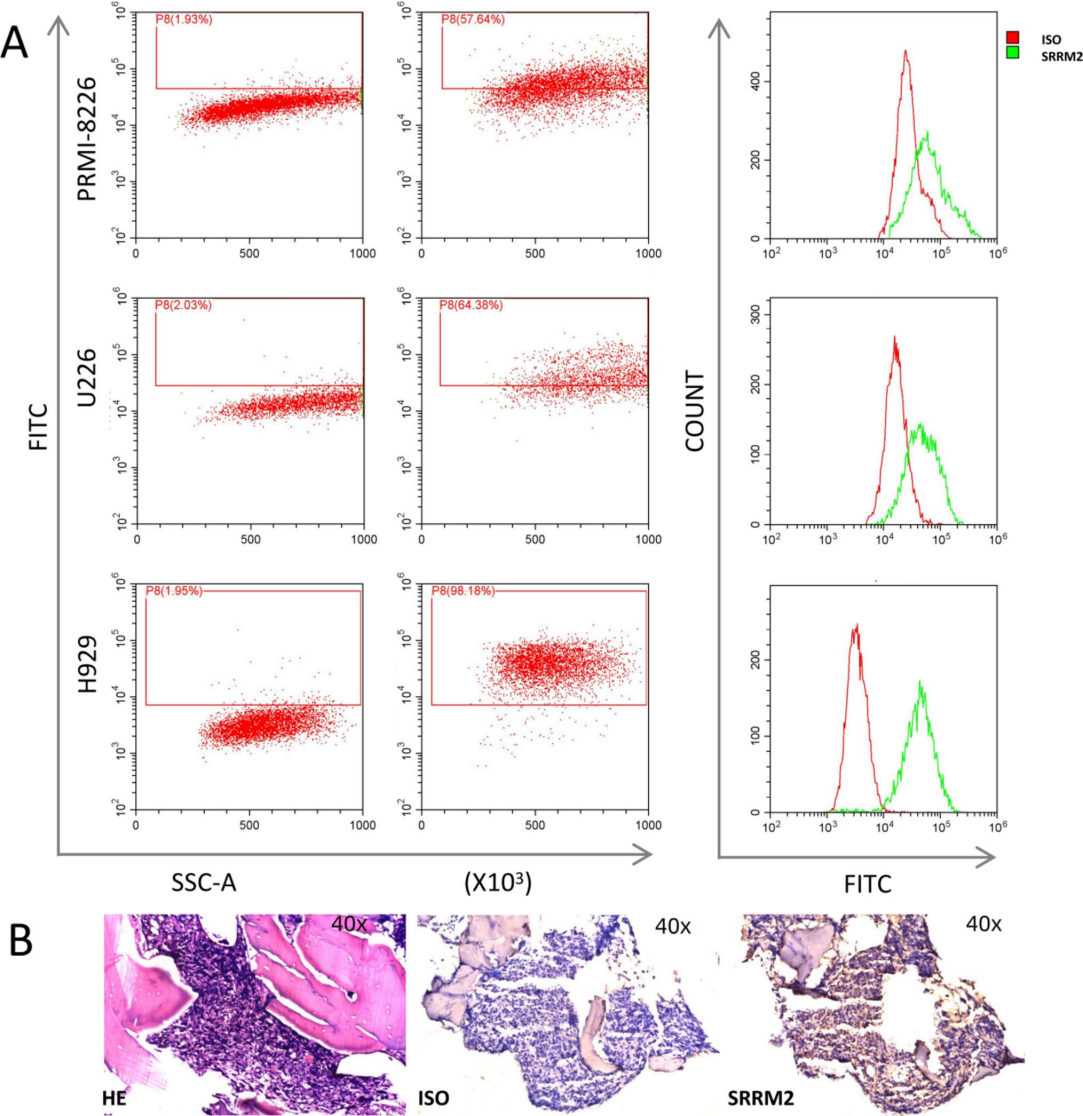
SRRM2 expression on multiple myeloma plasma cells. **A** Expression of SRRM2 on RPMI-8226, U226, and H929 cell lines, respectively. **B** Representative MM bone marrow sections were stained for HE and single immunoperoxidase labeling using anti-SRRM2 monoclonal antibody or ISO, respectively. All images were magnified 40 x

We detected the expression of SRRM2 in three human MM cell lines using flow cytometry and examined the bone marrow tissue of some clinical MM patients using immunohistochemistry. The results showed a positive expression of SRRM2 on the cell surface of all three MM cell lines. The expression rates of SRRM2 in RPMI-8226, U226, and H929 cell lines were 57.64%, 64.38%, and 98.18% (Fig. 5A), respectively. Immunohistochemical staining indicated high SRRM2 expression in the bone marrow of MM patients with high plasma cell infiltration (Fig. 5B).

### SRRM2 expression is higher on abnormal plasma cells

Patients with MM were divided into subgroups according to patient status at the time of testing and their response to the treatment: 29 of 35 newly diagnosed MM had positive SRRM2 expression on aberrant plasma cells, with a positive rate of 82.9%; 4 of 5 (VGPR + PR) subgroup samples had positive SRRM2 expression on aberrant plasma cells, with a positive rate of 80%; in the 28 cases (SD + PD) subgroup, 26 cases (92.9%) expressed SRRM2 positively on aberrant plasma cells; the expression of SRRM2 on aberrant plasma cells was positive in all 11 PCL subgroups, and the positive rate was 100%; SRRM2 was positively expressed on normal plasma cells in 2 of the 9 CR subgroup samples, with a positive rate of 22.2%. In addition, in the reactive plasmacytosis group, 4 of the 7 samples were positive for SRRM2 expression on normal plasma cells, with a positive rate of 57.1%; 6 of the 8 samples in other plasma cell disorders were positive for SRRM2 expression on aberrant plasma cells, with a positive rate of 75%. The distribution of positive expression of SRRM2 on plasma cells of various subgroups of plasmacytosis is shown in Fig. S1. In samples from patients with MM, all samples were negative for SRRM2 expression on granulocytes, monocytes, and lymphocytes in the newly diagnosed subgroup, the (VGPR + PR) subgroup, the PCL subgroup, and the CR subgroup. In the (SD + PD) subgroup, one granulocyte, five monocytes, and one lymphocyte expressed SRRM2 in 28 samples. Among the seven reactive plasmacytosis samples, one was positive for SRRM2 expression on granulocytes and monocytes. We also recorded the mean fluorescence intensity (MFI) of SRRM2 expression in plasma cells for each subgroup but observed no significant differences in expression levels between groups, and the results are summarized in Table S2.

**Fig. 6:**
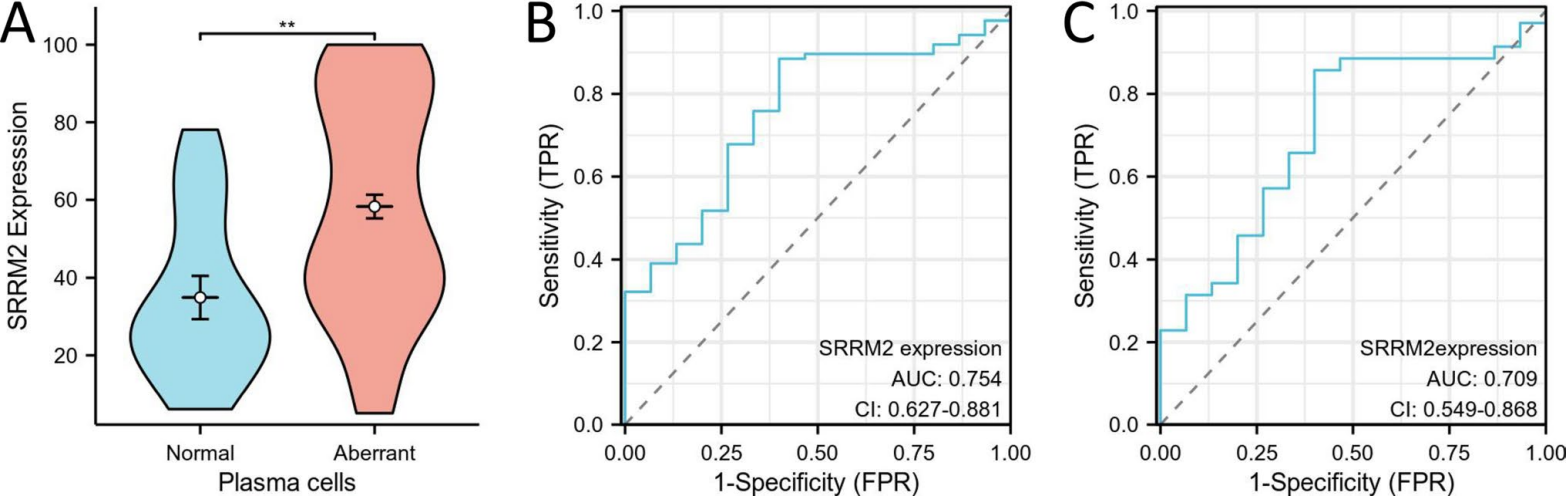
SRRM2 expression was increased on aberrant plasma cells. **A** Differential expression of SRRM2 on normal and abnormal plasma cells. **B** ROC curves of SRRM2 expression levels on aberrant plasma cells in Plasma cell dyscrasias. **C** ROC curves of SRRM2 expression levels on abnormal plasma cells in newly diagnosed MM. * P < 0.05, ** P < 0.01, *** P < 0.001, **** P < 0.0001

Next, we analyzed the differences in SRRM2 expression on normal plasma cells in the reactive plasmacytosis group and in the CR subgroup of MM and abnormal plasma cells in other subgroups of MM and other plasma cell dyscrasias (Fig. 6A) and found that SRRM2 expression was significantly elevated on abnormal plasma cells. The area under the curve for SRRM2 levels on abnormal plasma cells was determined by ROC curve analysis to be 0.75 (95% confidence interval, 0.63–0.88, *P* = 0.008). The maximum value of the Youden index (sensitivity + specificity-1) was 0.49, corresponding to 28.31% SRRM2 expression (Fig. 6B). The area under the curve of SRRM2 levels on newly diagnosed MM abnormal plasma cells was determined using ROC curve analysis to be 0.71 (95% confidence interval, 0.55–0.87, *P* = 0.02). The maximum Youden index value (sensitivity + specificity-1) was 0.46, which corresponded to 28.31% SRRM2 expression (Fig. 6C).

### SRRM2 is generally positively expressed on plasma cells, but rarely expressed on other normal blood cells

**Fig. 7:**
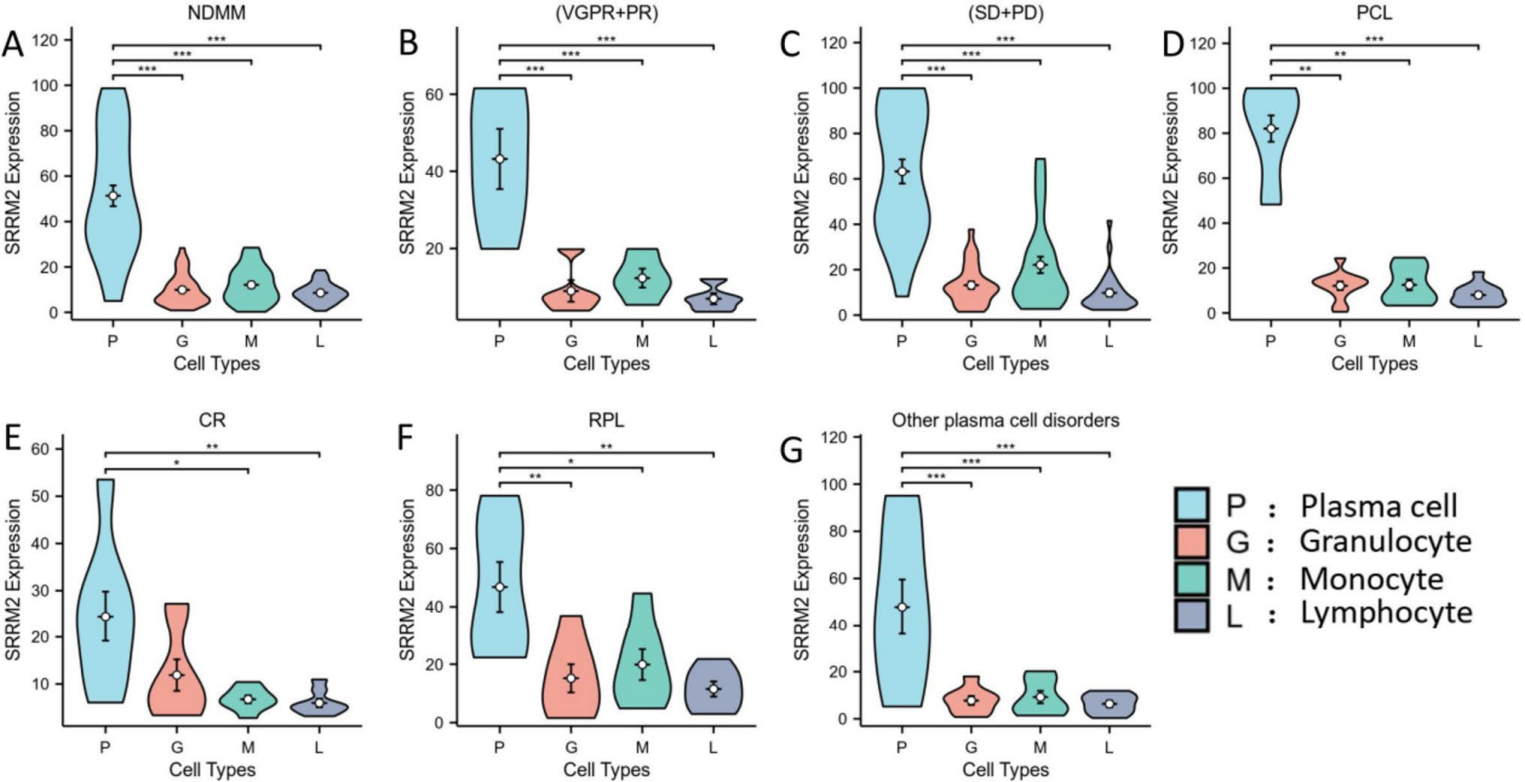
Differences in SRRM2 expression on plasma cells, granulocytes, lymphocytes, and monocytes in various subgroups of MM and other plasma cell dyscrasias. **A, B, C, D, E** Comparison of SRRM2 expression on plasma cells and other blood cells in various subgroups of MM. **F** Comparison of SRRM2 expression on plasma cells and other blood cells in reactive plasmacytosis. **G** Comparison of SRRM2 expression on plasma cells and normal blood cells in other plasma cell diseases. * P < 0.05, ** P < 0.01, *** P < 0.001, **** P < 0.0001. NDMM, Newly diagnosed multiple myeloma; PCL, plasmacytic leukemia; and RPL: reactive plasmacytosis subgroup

The expression of SRRM2 on plasma cells, granulocytes, monocytes, and lymphocytes was compared in each subgroup of plasma cell dyscrasias. The results showed that SRRM2 expression on plasma cells was significantly higher than that on normal blood cells in all subgroups, including MM, reactive plasmacytosis, and other plasma cell dyscrasias. As shown in Table S2 and Fig. 7, in all subgroups of plasma cell dyscrasias, the expression of SRRM2 on granulocytes, monocytes, and lymphocytes was low and mostly negative; however, in the subgroup of MM at (SD + PD) stage, we found an increased expression of SRRM2 on granulocytes and monocytes and an increased positive rate, which was still lower than the expression of SRRM2 on plasma cells, and the difference was statistically significant. Meanwhile, we found that in the CR subgroup of MM, although the expression of SRRM2 on plasma cells was higher than that on other normal blood cells, the difference between SRRM2 expression on plasma cells and granulocytes was not statistically significant.

### Relationship between SRRM2 expression on plasma cells and patient clinical profiles in patients with newly diagnosed MM

Patients with newly diagnosed MM were categorized into two groups, based on the level of SRRM2 expression on plasma cells: SRRM2-negative (SRRM2 expression < 30%, *n* = 6) and SRRM2-positive (SRRM2 expression ≥ 30%, *n* = 29). Patients in the SRRM2-negative group were more likely to exhibit lower levels of β2-microglobulin (β2-MG), lower percentages of bone marrow plasma cells (BMPC), lower percentages of bone marrow plasma cells counted by flow cytometry (F-BMPC), lower serum levels of lactate dehydrogenase (LDH), be in ISS stages I and II, have a standard-risk mSMART 3.0 risk stratification, and exhibit standard-risk cytogenetic abnormalities. Our study also revealed no significant differences between the two groups in terms of age, sex, monoclonal globulin type, hemoglobin level, platelet count, erythrocyte distribution width (RDW), granulocyte-to-lymphocyte ratio (NLR), platelet-to-lymphocyte ratio (PLR), lymphocyte-to-monocyte ratio (LMR), systemic immune inflammatory index (SII), systemic inflammatory response index (SIRI), albumin, tests related to renal function, serum calcium and phosphorus levels, iron metabolism testing, folate and vitamin B12 levels, CD56 expression in plasma cells, D-S stage, R-ISS stage, extramedullary infiltration of myeloma, and bone destruction (Table 1).

**Table 1:** Relationship between SRRM2 expression levels on plasma cells and clinicopathological features of newly diagnosed MM

Characteristic		All cases (*n* = 35)	SRRM2 negative(*n* = 6)	SRRM2 positive(*n* = 29)	*P* value
Age, median (range) years		63(35–84)	62(49–75)	63(35–84)	0.844
Age group, years	≥ 65	17	5	22	1
	< 65	18	1	7	
Gender	Female	18	1	17	0.088
	Male	17	5	12	
Type	IgG	16	5	11	0.141
	IgA	6	0	6	
	IgD	2	0	2	
	Light chain	11	1	10	
Light chain type	κ	14	4	10	0.191
	λ	21	2	19	
Hb (g/L)	≥ 85	15	2	20	0.166
	< 85	20	4	9	
PLT (× 109/L)	≥ 100	29	6	23	0.561
	< 100	6	0	6	
NLR, median (range)		2.15(0.79–5.96)	2.26(0.79–5.96)	2.15(1.07–3.96)	0.765
PLR, median (range)		114.6(38.83–336.49)	108.52(59.17–273.96)	117.06(38.84–336.49)	0.848
LMR, median (range)		3.65(0.73–11.27)	4.28(1.8–6.67)	3.38(0.73–11.27)	0.379
SII, median (range)		290.4(97.73–1061.4)	288.99(112.42–1061.4)	334.24(97.73–910.82)	0.729
SIRI, median (range)		0.87(0.2–4.02)	0.71(0.22–2.68)	0.94(0.2–4.02)	0.676
ALB (g/L)	≥ 35	14	1	13	0.366
	< 35	21	5	16	
Cr (μmol/L)	≥ 117	13	1	12	0.377
	< 117	22	5	17	
BUN (mmol/L)	> 8	19	2	17	0.379
	≤ 8	16	4	12	
UA(μmol/L)	> 420	16	2	14	0.666
	≤ 420	19	4	15	
RDW, median (range) %		14.4(12.2–24.2)	14.3(13.5–18.4)	14.3(12.2–24.2)	0.948
P (mmol/L)	> 1.51	16	2	14	0.666
	≤ 1.51	19	4	15	
Ca^2+^ (mmol/L)	> 2.65	5	0	5	0.561
	≤ 2.65	30	6	24	
Fe (μmol/L)	≥ 10.6	15	3	12	1
	< 10.6	11	2	9	
	NA	9	1	8	
FEP, median (range) μg/L		372.5(8.9–25,900)	378(8.9–25,900)	367(279–3040)	0.474
	NA	9	1	8	
TFP, median (range) g/L		1.7(1–2.6)	1.5(1.3–1.7)	1.9(1–2.6)	0.167
	NA	9	1	8	
Folate, median (range) nmol/L		16.55(4.28–50.1)	12.5(6.5–18.8)	16.55(4.28–50.1)	0.462
	NA	11	2	9	
VitB12, median (range) pmol/L		177(111–575)	145.5(111–530)	177.5(111–575)	0.612
	NA	11	2	9	
β2-MG (mg/L)	> 4.5	25	2	23	0.043
	≤ 4.5	10	4	6	
β2-MG, median (range) mg/L		7.02(2–65.6)	4.43(3.76–7.02)	8.3(2–65.6)	0.035
CD56 expression	negative	12	3	9	0.391
	positive	23	3	20	
BMPC %	≥ 20	22	1	21	0.019
	< 20	13	5	8	
BMPC median (range) %		28(3–83.5)	13.5(3–49)	30(5.5–83.5)	0.023
F-BMPC %	≥ 10		1	23	0.007
	< 10		5	6	
F-BMPC, median (range) %		18(2.4–62)	6.65(2.4–17)	24(3.3–6.2)	0.009
D-S Stage	IA&IB + IIA&IIB	4	2	2	0.128
	IIIA&IIIB	31	4	27	
ISS Stage	I + II	14	5	9	0.028
	III	21	1	20	
R-ISS Stage	I + II	15	5	10	0.169
	III	14	1	13	
	NA	6	0	6	
mSMART 3.0	High-Risk	21	1	20	0.001
	Standard-Risk	7	5	2	
	NA	7	0	7	
Cytogenetics abnormalities	High-Risk	19	1	18	0.007
	Standard-Risk	9	5	4	
	NA	7	0	7	
CRP (mg/L)	> 5	12	1	11	0.640
	≤ 5	23	5	18	
CRP median (range) mg/L		2.25(0.2–56.3)	1.95(0.2–5.1)	2.25(0.27–56.3)	0.229
LDH (U/L)	> 250		0	7	0.311
	≤ 250		6	22	
LDH median (range) U/L		163(91–559)	135.5(91–184)	165(105–559)	0.035
Extramedullary myeloma	YES	5	1	4	1
	NO	30	5	25	
Bone destruction	≥ 3		2	21	0.151
	< 3		4	8	

Hb, Hemoglobin; PLT, Platelets; NLR, granulocyte-to-lymphocyte ratio; PLR, platelet-to-lymphocyte ratio; LMR, lymphocyte-to-monocyte ratio; SII, systemic immune inflammatory index; SIRI, systemic inflammatory response index; ALB, Albumin; Cr, Creatinine; BUN, Urea nitrogen; UA, Uric acid; RDW, Red blood cell distribution width; P, Serum phosphorus; Ca2 + , Blood Calcium; Fe, Serum iron; FEP, Ferritin; TFP, Transferrin; β2-MG, β2 microglobulin; BMPC, bone marrow plasma cells; F-BMPC, bone marrow plasma cells counted by flow cytometry; CRP, c-reactive protein; and LDH, Lactate dehydrogenase

**Fig. 8:**
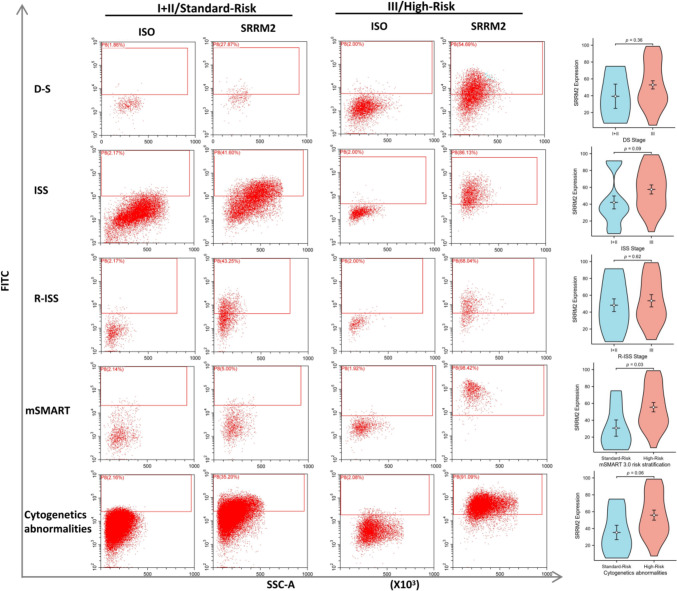
Differences in SRRM2 expression on plasma cells in newly diagnosed MM in different stages, prognostic stratification systems, and cytogenetic abnormalities, as well as illustrations showing representative flow cytometry analysis of each group

We compared SRRM2 expression on plasma cells with different DS, ISS, R-ISS, mSMART 3.0, and cytogenetic abnormalities (Fig. 8). The results showed significantly higher SRRM2 expression on plasma cells of MM patients with high-risk mSMART 3.0 stratification, and the difference was statistically significant. Patients with MM with higher ISS staging and high-risk cytogenetic abnormalities also showed higher SRRM2 expression on plasma cells. The differences in SRRM2 expression on plasma cells in patients with MM with different DS and R-ISS staging were not significant.

Thirty-five newly diagnosed with MM were analyzed, of whom 28 underwent FISH analysis for cytogenetics. Among them, 1q21 amplification was detected in 14 cases, P53 (17p13) deletion in 4 cases, t(4;14) translocation in 4 cases, and t(14;16) and t(14;20) translocations in each case, while nine cases had no high-risk cytogenetic abnormalities. Among MM patients with high-risk cytogenetic abnormalities, two had 1q21 amplification accompanied by t(14;20) translocation, one had 1q21 amplification accompanied by t(14;16) translocation, and one had 1q21 amplification accompanied by t(14;16) translocation and P53 deletion. We also grouped the patients based on positive or negative expression of SRRM2 in plasma cells and analyzed the relationship between each high-risk cytogenetic abnormality and SRRM2 expression. The results showed that the SRRM2-positive expression group was more likely to exhibit 1q21 amplification (Table S3).

### The relationship between SRRM2 expression on previously diagnosed MM plasma cells and patient clinical profiles

We also analyzed the relationship between SRRM2 expression on previously diagnosed MM plasma cells and the clinical profiles of patients (Table 2). Previously diagnosed MM was divided into four subgroups according to treatment response and disease status: CR, (VGPR + PR), (SD + PD), and PCL. Of the 11 PCL samples tested, five were progressive from the (SD + PD) subgroup. The test data and clinical data of these five patients were collected for inclusion in this analysis when the patients were PCL. Our analysis suggests that patients in the SRRM2-negative group were more likely to have standard-risk mSMART 3.0 risk stratification, more autologous stem cell transplantation treatments, lower percentages of bone marrow plasma cells counted by flow cytometry (F-BMPC), and fewer relapses. Our analysis also showed no differences between the two groups in terms of age, sex, monoclonal globulin type, light chain type, D-S stage, ISS stage, R-ISS stage, cytogenetic abnormalities, extramedullary myeloma, hepatitis B infection, occurrence of gastritis/gastric ulcer, and thyroid function abnormalities. The main thyroid function abnormalities detected include hypothyroidism, low T3 syndrome, and other euthyroid syndromes.

**Table 2:** Relationship between SRRM2 expression on previously diagnosed MM plasma cells and the patient’s clinical profile

Characteristic		All cases (*n* = 47)	SRRM2 negative(*n* = 8)	SRRM2 positive(*n* = 39)	*P* value
Age, median (range) years		64(39–81)	62(45–73)	64(39–81)	0.815
Age group, years	≥ 65 years	21	3	18	0.653
	< 65 years	26	5	21	
Gender	Female	18	3	15	0.959
	Male	29	5	24	
Type	IgG	20	1	19	0.101
	IgA	11	1	10	
	IgD	5	0	5	
	Light chain	10	5	5	
	Dual Cloning	1	1	0	
Light chain type	κ	21	4	17	0.74
	λ	26	4	22	
F-BMPC %	≥ 10	21	1	20	0.044
	< 10	26	7	19	
F-BMPC, median (range) %		8.6(0.1–77)	0.65(0.1–50)	10(0.2–77)	0.034
D-S Stage	IA&IB + IIA&IIB	5	1	4	0.851
	IIIA&IIIB	42	7	35	
ISS Stage	I + II	22	4	18	0.843
	III	25	4	21	
R-ISS Stage	I + II	22	6	16	0.116
	III	15	1	14	
	NA	10	1	9	
mSMART 3.0	High-Risk	23	2	21	0.042
	Standard-Risk	14	5	9	
	NA	10	1	9	
Cytogenetics abnormalities	High-Risk	19	2	19	0.095
	Standard-Risk	9	5	11	
	NA	10	1	9	
Extramedullary myeloma	YES	6	1	5	0.98
	NO	41	7	34	
Hepatitis B	YES	7	1	6	0.835
	NO	40	7	33	
Gastritis/gastric ulcer	YES	9	1	8	0.6
	NO	38	7	31	
Herpes	YES	4	1	3	0.657
	NO	43	7	36	
Thyroid function	Normal	18	4	14	0.484
	Anomalies	9	1	8	
	NA	20	3	17	
Autologous transplant	YES	7	3	4	0.049
	NO	40	5	35	
Recurrence	≥ 1	22	1	31	0.001
	< 1	25	7	8	

**Fig. 9:**
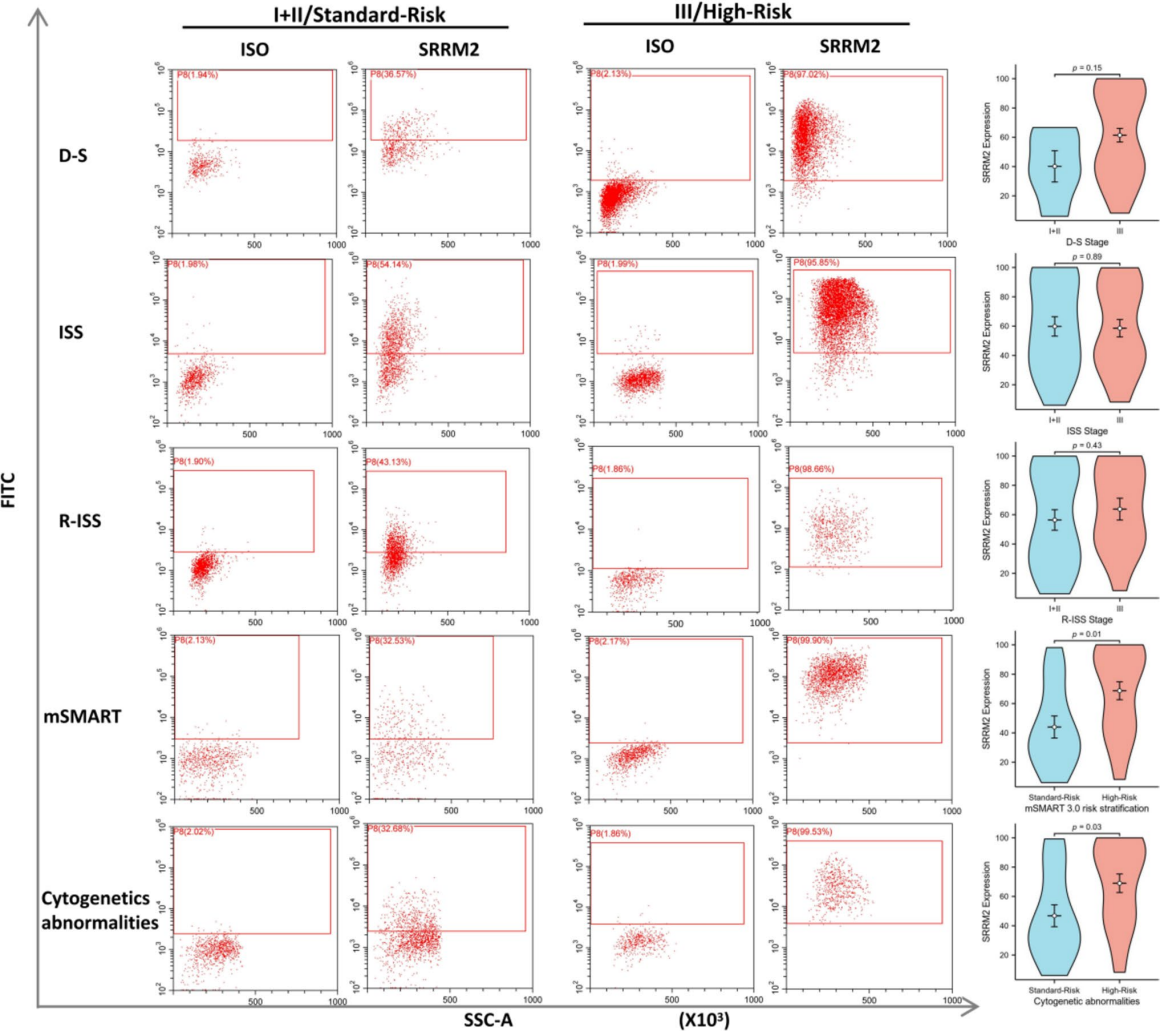
Differences in SRRM2 expression on plasma cells in previously diagnosed MM in different stages, prognostic stratification systems, and cytogenetic abnormalities, as well as illustrations showing representative flow cytometry analysis of each group

Cytogenetic FISH testing was performed on 37 of the 47 patients with previously diagnosed MM. Among them, 1q21 amplification was detected in 17 cases, P53 deletion was detected in three cases, t(4;14) translocation in five cases, t(14;16) translocation in two cases, and non-high-risk karyotype abnormalities in 16 cases. Among the patients with high-risk karyotype abnormalities, three cases of 1q21 amplification with t(4;14) translocation, one case of 1q21 amplification with t(4;16) translocation, and one case of 1q21 amplification with t(4;14) translocation and P53 deletion were detected. Similarly, we compared SRRM2 expression on previously diagnosed MM plasma cells with different DS, ISS, R-ISS, mSMART 3.0, and cytogenetic abnormalities (Fig. 9). The results showed that SRRM2 expression was higher on MM plasma cells in mSMART 3.0, high-risk stratification, and in the presence of high-risk cytogenetic abnormalities, and the difference was statistically significant.

### The expression of SRRM2 in plasma cells is related to the treatment response of MM, or may have the potential to prognosis of MM

**Fig. 10:**
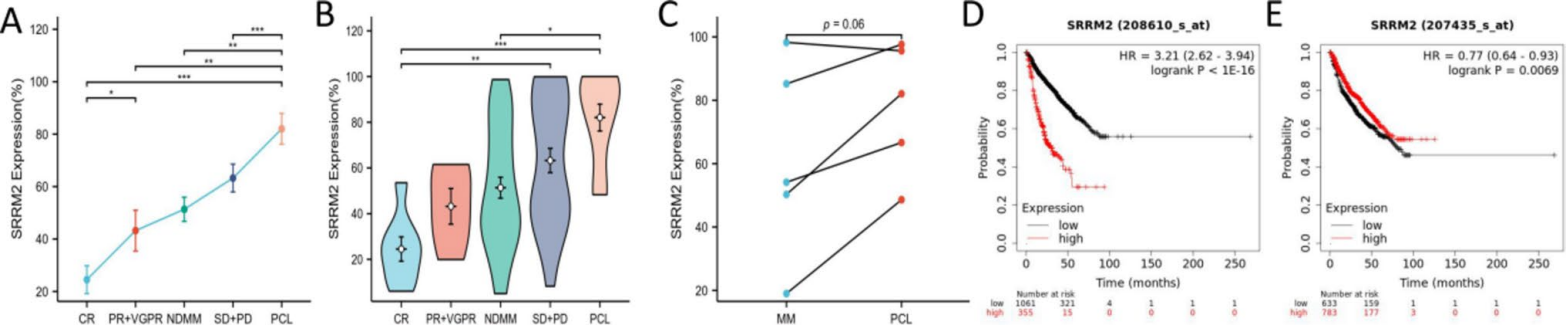
The expression of SRRM2 in plasma cells is associated with the disease status and treatment response of MM. **A** Folding line graph of SRRM2 expression on plasma cells under different disease states and treatment responses in MM. **B** Differences in SRRM2 expression on plasma cells under different disease states and treatment responses in MM. **C** MM patients who progressed to PCL showed an increase in SRRM2 expression on their plasma cells. **D, E** The relationship between SRRM2 expression at the transcriptional level and overall survival in different datasets of MM patients was examined in a Kaplan–Meier plotter

Finally, we investigated the association between the expression of SRRM2 on plasma cells and different subgroups of MM. Patients with MM who were followed up were divided into five subgroups according to their disease status and treatment response: CR, (VGPR + PR), NDMM, (SD + PD), and PCL. As shown in the line graph of Fig. 10A, the expression of SRRM2 on plasma cells showed a significant increase from CR, (VGPR + PR), NDMM, (SD + PD) to PCL subgroups. As shown in Fig. 10B, the subgroups of PCL and progressive relapsed MM exhibited significantly higher levels of SRRM2 expression on plasma cells than those in remission from MM. In addition, PCL showed significantly higher SRRM2 expression on plasma cells than in newly diagnosed MM. All of these differences were statistically significant. The above analysis indicates that through follow-up testing of 71 samples from 64 patients with MM, SRRM2 expression on plasma cells in MM was correlated with the response to treatment of the disease. The expression of plasma cell SRRM2 significantly increased in relapsed progressive MM and PCL. In addition, in follow-up monitoring, five MM patients progressed to PCL, and we compared the expression levels of SRRM2 in plasma cells before and after disease progression in these five patients. The results showed that the expression of SRRM2 in plasma cells had an increasing trend during the progression from MM to PCL (Fig. 10C). This suggests that the dynamic increase in SRRM2 expression in plasma cells may be associated with disease progression. Although the follow-up time of clinical patients is limited and the survival prognosis of patients cannot be analyzed, we tried to analyze the relationship between SRRM2 at the gene level and the survival prognosis of MM patients through public data sets. Interestingly, Kaplan–Meier plotter analysis suggested that SRRM2 expression showed different or even opposite correlations with MM prognosis in different data sets (Fig. 10D, E).

## Discussion

SRRM2, as a marker of nuclear speckles, is an important splicing factor in spliceosomal structures, and its dysfunction and mislocalization are associated with various human diseases [19–22]. This study provides evidence for SRRM2 expression on the surface of aberrant plasma cell membranes by designing a flow cytometry protocol to detect SRRM2 expression in MM cell lines and on the surface of plasma cells and other normal blood cells during plasma cell dyscrasias. In this study, we found that SRRM2 is expressed on the surface of plasma membranes in various MM cell lines. Clinical testing of patients showed high expression of SRRM2 on plasma cells in various plasma cell dyscrasias, including reactive plasma cells, while its expression on other normal blood cells is rare. Furthermore, our research demonstrated that SRRM2 expression was significantly higher on abnormal plasma cells, such as those in MM, than on normal plasma cells, such as reactive plasma cells. High expression of SRRM2 on plasma cells has diagnostic value in clonal plasma cell dyscrasias and newly diagnosed MM. Additionally, in cases of clonal proliferation of plasma cells, such as in MM, SRRM2 is rarely expressed on other normal blood cells, including granulocytes, monocytes, and lymphocytes. These findings suggest that SRRM2 may be a promising target antigen for novel immunotherapeutic approaches for MM, such as immunoglobulin-based therapies or immune cell therapies, like CAR-T cell therapy. Current data from MM cell lines and clinical patient samples provide fundamental real-world evidence of the feasibility and safety of targeting SRRM2 for immunotherapy in MM.

High expression of SRRM2 in plasma cells of newly diagnosed MM patients is associated with poor prognosis indicators, including elevated levels of serum β2-MG and LDH, higher ISS stage, increased risk of high-risk mSMART 3.0 risk stratification, and increased risk of cytogenetic abnormalities. This suggests that SRRM2 expression may be prognostic in MM, although longer follow-up is needed to determine its association with progression-free survival (PFS) and overall survival (OS). Elevated bone marrow infiltration was also observed in patients with high SRRM2 expression, indicating that SRRM2 may affect the activity and proliferation of MM plasma cells. Moreover, MM patients with SRRM2 positive expression on plasma cells had a higher incidence of 1q21 amplification, which is a common secondary cytogenetic abnormality associated with poor prognosis, drug resistance, and disease progression [23]. However, the specific genes driving high-risk disease progression associated with 1q21 amplification have not been fully identified yet [24]. SRRM2, as an important splicing factor in splicing complex structures, may play a role in 1q21 amplification when its function is impaired or mislocalized, which needs further investigation.

The severity of anemia and hypercalcemia are poor prognostic factors for MM, and there is also evidence that inflammatory markers and some iron metabolism-related markers are associated with prognosis in MM [25–29]. We investigated the correlation between SRRM2 expression in plasma cells and various inflammatory markers such as NLR, PLR, LMR, SII, SIRI, RDW, and CRP, iron metabolism-related markers, and markers of calcium-phosphate metabolism and kidney function in newly diagnosed MM patients. However, we observed no significant differences in the expression of these markers between the SRRM2-negative and SRRM2-positive groups, indicating that SRRM2 may not be strongly associated with these factors in the development and progression of MM. Our study results suggest that the prognostic relevance of SRRM2 in MM may have some limitations due to the reasons mentioned above.

In addition, analysis of MM during treatment has shown that despite the influence of different treatment plans and response stages, MM patients with high expression of plasma cell SRRM2 still exhibit higher mSMART 3.0 risk stratification and cytogenetic abnormality risk. The consistency between plasma cell SRRM2 expression and mSMART 3.0 risk stratification and cytogenetic abnormalities suggests that plasma cell SRRM2 expression could even serve as a good indicator for MM risk stratification. Despite the influence of treatment, MM patients with high SRRM2 expression still showed a higher proportion of plasma cell infiltration, indicating that SRRM2 expression in plasma cells may affect their proliferation and invasion. Our analysis also showed that patients with MM who received autologous stem cell transplantation had lower levels of SRRM2 expression in plasma cells. Furthermore, high plasma cell SRRM2 expression was associated with a higher number of relapses in previously diagnosed MM patients, suggesting that refractory relapsed MM may have high plasma cell SRRM2 expression.

Compared with patients with MM at diagnosis, most patients with MM who achieve CR, VGPR, or PR show decreased levels of plasma cell SRRM2. Conversely, higher levels of SRRM2 were detected in the plasma cells of patients with PD relapse and PCL. The expression level of SRRM2 in plasma cells of MM patients may be associated with disease relapse and progression, and patients with high expression of SRRM2 in plasma cells may be more prone to transformation into PCL. Follow-up of MM patients who progress to PCL revealed an upward trend in SRRM2 expression on plasma cells, further demonstrating the relationship between SRRM2 expression and disease progression as well as plasma cell invasive properties in MM. Finally, we showed through public data set analysis that the expression of SRRM2 at the gene level shows different or even opposite correlations with MM prognosis in different data sets. This indicates that the high expression of SRRM2 on plasma cells may not be caused by high expression at the gene level. We speculate that in MM, abnormal localization of nuclear export of SRRM2 in abnormally proliferating clonal plasma cells may occur through certain mechanisms, which requires further mechanistic exploration.

## Conclusion

In summary, we demonstrated that SRRM2 is a novel biomarker for MM and has the potential to serve as a target for immunotherapy in this disease. The expression level of SRRM2 on plasma cells can aid in risk stratification and monitoring of treatment responses in MM. Additionally, we hypothesize that SRRM2 expression may be associated with the prognosis of MM and the proliferation and invasive properties of plasma cells. We look forward to multicenter verification of this finding and at the same time carry out in vivo and in vitro studies to further explore the biological function and clinical significance of SRRM2 expression on plasma cells.

## Supplementary Information

Below is the link to the electronic supplementary material.

Supplementary file1 (DOCX 97 KB)

## Data Availability

The data that support the findings of this study are available from the corresponding author (ZM.Z) upon reasonable request.
