# Correction to: SRRM2 may be a potential biomarker and immunotherapy target for multiple myeloma: a real-world study based on flow cytometry detection

**DOI:** 10.1007/s10238-024-01393-1

**Published:** 2024-07-18

**Authors:** Jinjing Guo, Zhiye Zhang, Huiping Wang, Qian Li, Mengmeng Fan, Wanqiu Zhang, Qianshan Tao, Zhitao Wang, Chun Ling, Hao Xiao, Zhimai Gao, Zhimin Zhai

**Affiliations:** 1 Department of Hematology, Hematological Research Center, The Second Affiliated Hospital of Anhui Medical University, Hefei, Anhui China; 2 Department of Laboratory, Fuyang People’s Hospital, Fuyang, China; 3 Department of Hematology, Fuyang Hospital Affiliated to Anhui Medical University, Fuyang, China; 4 Department of Hematology, Affiliated Chuzhou Hospital of Anhui Medical University, Chuzhou, Anhui China; 5 ZENO Biotechnology (Shenzhen) Co, Shenzhen, Guangzhou, China

**Correction to: Clinical and Experimental Medicine (2024) 24:28** 10.1007/s10238-023-01272-1

In the original version of this article, Acknowledgements section was incorrect. The acknowledgements section which previously read

The authors would like to thank Dr. Lietao Li and Dr. Tielin Zhou (ZENO THERAPEUTICS PTE. LTD.) for generously providing the ISO and SRRM2 antibodies and technical support. We thank Zhenzhong Feng, Panpan Yang (Pathology Center, Second Affiliated Hospital of Anhui Medical University), and Yang Wan (Laboratory of Hematology, Second Affiliated Hospital of Anhui Medical University) for their help in the preparation and analysis of pathological sections.

should have read

The authors would like to express their sincere gratitude to Professor Reinhard Zeidler and his research group (Research Unit Gene Vectors, Helmholtz Center Munich, German Research Center for Environmental Health; Department of Otorhinolaryngology, Klinikum der Universität) for developing specific antibodies against SRRM2 and for being the first to propose that SRRM2 may be expressed in some tumor cells.

