# Supplementary material for: SRRM2 may be a potential biomarker and immunotherapy target for multiple myeloma: a real-world study based on flow cytometry detection
Source: Clin Exp Med. 2024 Jan 30;24(1):28. doi: 10.1007/s10238-023-01272-1 (PMC10827842; doi:10.1007/s10238-023-01272-1)
Supplement: Supplementary file 1 — Supplementary file1 (DOCX 97 KB) [file 10238_2023_1272_MOESM1_ESM.docx]

**Supplementary Data**

**Supplementary Figures**

**Figure S1.** Expression of SRRM2 on plasma cells in various subgroups of plasma cell

dyscrasias.

**Supplementary Tables**

**Table S1.** Baseline characteristics of the study group.

**Table S2.** Expression of SRRM2 on plasma cells and other blood cells in plasma cell

disease.

**Table S3.** Relationship between SRRM2 expression levels on plasma cells and

cytogenetics abnormalities of newly diagnosed MM.

**
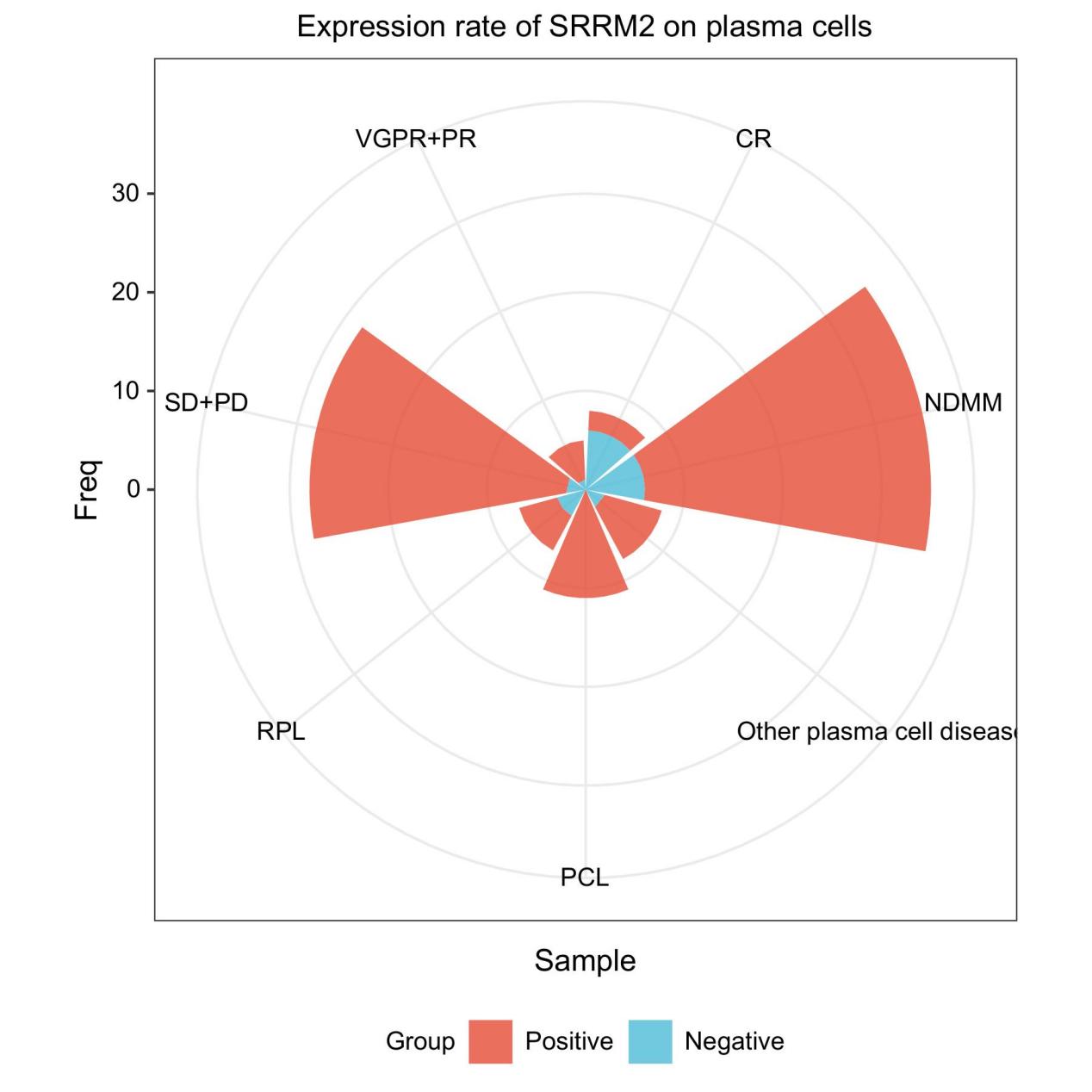
**

**Figure S1. Expression of SRRM2 on plasma cells in various subgroups of plasma**

**cell dyscrasias .**

SRRM2 was commonly positively expressed on plasma cells from all subgroups of

plasma cell dyscrasias.

**Abbreviations:** NDMM, Newly diagnosed multiple myeloma; PCL, plasmacytic

leukemia; RPL: Reactive plasmacytosis; CR:complete response; PR: partial response;

VGPR: very good partial response; SD: stable disease; PD: disease progression.

**Table S1. Baseline characteristics of the study group.**

| **Patient characteristics** | **MM (n=80)** | **Reactive plasmacytosis (n=7)** | **Other plasma cell disorders (n=8)** |
| --- | --- | --- | --- |
| Age, median (range) years | 64(35-84) | 70(38-82) | 67(52-75) |
| Gender (female/male) | 35/45 | 3/4 | 0/8 |
| Type (IgG/IgA/IgD/ Light chain/Dual Cloning) | 35/17/6/21/1 | NA | NA |
| Light chain type (κ/λ) | 35/45 | NA | NA |
| Renal function (A-Normal/B-Abnormal) | 54/26 | NA | NA |
| Durie-Salmon stage (I/II/III) | 2/7/71 | NA | NA |
| ISS stage (I/II/III) | 5/31/44 | NA | NA |
| R-ISS stage (I/II/III/NA) | 4/32/28/16 | NA | NA |
| mSMART 3.0 risk (High/Standard/NA) | 21/42/17 | NA | NA |
| 1q21 amplification (Present/Absent/NA) | 31/32/17 | NA | NA |
| P53 deletion (Present/Absent/NA) | 7/56/17 | NA | NA |
| t(4;14) (Present/Absent/NA) | 9/54/17 | NA | NA |
| t(14;16) Present/Absent/NA) | 3/60/17 | NA | NA |
| t(14;20) (Present/Absent/NA) | 1/62/17 | NA | NA |

**Table S2. Expression of SRRM2 on plasma cells and other blood cells in plasma cell dyscrasias.**

| **Types of plasma cell dyscrasias** | **Subgroup** | **Cell type** | **No. Cases** | **PositiveCases** | **%PositiveCases** | **MFI median (range)** |
| --- | --- | --- | --- | --- | --- | --- |
| Reactive plasmacytosis |  | Plasma cell | 7 | 4 | 57.1 | 35586(11501-75706) |
|  |  | Granulocyte | 7 | 1 | 14.3 |  |
|  |  | Monocyte | 7 | 1 | 14.3 |  |
|  |  | Lymphocyte | 7 | 0 | 0 |  |
| Multiple myeloma | NDMM group | Plasma cell | 35 | 29 | 82.9 | 37095(6945-312905) |
|  |  | Granulocyte | 35 | 0 | 0 |  |
|  |  | Monocyte | 35 | 0 | 0 |  |
|  |  | Lymphocyte | 35 | 0 | 0 |  |
|  | (VGPR+PR) group | Plasma cell | 5 | 4 | 80 | 29052(13531-56413) |
|  |  | Granulocyte | 5 | 0 | 0 |  |
|  |  | Monocyte | 5 | 0 | 0 |  |
|  |  | Lymphocyte | 5 | 0 | 0 |  |
|  | (SD+PD) group | Plasma cell | 28 | 26 | 92.9 | 35944(3805-196131) |
|  |  | Granulocyte | 28 | 1 | 3.6 |  |
|  |  | Monocyte | 28 | 5 | 17.9 |  |
|  |  | Lymphocyte | 28 | 1 | 3.6 |  |
|  | PCL group | Plasma cell | 11 | 11 | 100 | 24281(3568-67676) |
|  |  | Granulocyte | 11 | 0 | 0 |  |
|  |  | Monocyte | 11 | 0 | 0 |  |
|  |  | Lymphocyte | 11 | 0 | 0 |  |
|  | CR group | Plasma cell | 9 | 2 | 22.2 | 37248(9559-125480) |
|  |  | Granulocyte | 9 | 0 | 0 |  |
|  |  | Monocyte | 9 | 0 | 0 |  |
|  |  | Lymphocyte | 9 | 0 | 0 |  |
| Other plasma cell disorders |  | Plasma cell | 8 | 6 | 75 | 33276(12424-63586) |
|  |  | Granulocyte | 8 | 0 | 0 |  |
|  |  | Monocyte | 8 | 0 | 0 |  |
|  |  | Lymphocyte | 8 | 0 | 0 |  |

Abbrebiations:NDMM, Newly diagnosed multiple myeloma; PCL, plasmacytic leukemia ; MFI: mean fluorescence intensity; CR:complete response; PR: partial response; VGPR: very good partial response; SD: stable disease; PD: disease progression.

**Table S3. Relationship between SRRM2 expression levels on plasma cells and cytogenetics abnormalities of newly diagnosed MM.**

| **Cytogenetic abnormalities** |  | **All cases （n=35）** | **SRRM2 negative（n=6）** | **SRRM2 positive（n=29）** | **P value** |
| --- | --- | --- | --- | --- | --- |
| 1q21 | YES | 14 | 0 | 14 | 0.016 |
|  | NO | 14 | 6 | 8 |  |
|  | NA | 7 | 0 | 7 |  |
| P53 | YES | 4 | 1 | 3 | 1 |
|  | NO | 24 | 5 | 19 |  |
|  | NA | 7 | 0 | 7 |  |
| t(4;14) | YES | 4 | 0 | 4 | 0.549 |
|  | NO | 14 | 6 | 18 |  |
|  | NA | 7 | 0 | 7 |  |
| t(14;16) | YES | 1 | 0 | 1 | 1 |
|  | NO | 27 | 6 | 21 |  |
|  | NA | 7 | 0 | 7 |  |
| t(14;20) | YES | 1 | 0 | 1 | 1 |
|  | NO | 27 | 6 | 21 |  |
|  | NA | 7 | 0 | 7 |  |
